# Still an Unsolved Question: The Place of Cranial Magnetic Resonance Imaging in Acute Acquired Concomitant Esotropia

**DOI:** 10.3390/children11050519

**Published:** 2024-04-26

**Authors:** Luisa Mittendorf, Matthias K. Bernhard, Ina Sterker, Wieland Kiess, Janina Gburek-Augustat, Andreas Merkenschlager

**Affiliations:** 1 Division of Neuropediatrics, Hospital for Children and Adolescents, Department of Women and Child Health, University of Leipzig, Liebigstraße 20a, 04103 Leipzig, Germany; matthias.bernhard@medizin.uni-leipzig.de (M.K.B.); andreas.merkenschlager@medizin.uni-leipzig.de (A.M.); 2 Department of Head and Dental Medicine, Hospital for Ophthalmology, University of Leipzig, Liebigstraße 12, 04103 Leipzig, Germany

**Keywords:** strabismus, AACE, cMRI

## Abstract

Purpose: The aim of this study was to collect further data to estimate the risk of relevant intracranial pathology and thereby better assess the need for cranial imaging in children with acute acquired comitant esotropia (AACE). To date, there is still not enough literature on this topic to enable a consensus on the diagnostic algorithm. Methods: We analyzed data from patients with convergent strabismus who received cranial imaging via magnetic resonance imaging (MRI). Twenty-one patients received a cranial MRI for the diagnostic evaluation of AACE. The age range was from 2 to 12 years, and the mean age at the time of diagnosis was 5.5 years. Of these patients, only one exhibited insignificant MRI findings, with no therapeutic consequences. Conclusions: Our data add further evidence that AACE without neurological findings or other ophthalmologic anomalies might not be an indication for cranial MRI as a diagnostic screening tool.

## 1. Introduction

Childhood-onset strabismus is common in young infants. The prevalence of comitant strabismus is estimated to be between 2.3% and 6% [1]. In a cross-sectional population-based study of 12-year-old children in Australia, strabismus was found in 2.8% of children, with 54% exhibiting esotropia, 29% exotropia, and cranial nerve palsy in just one child. The risk of strabismus was higher in premature infants and when vision was impaired [2].

Acute strabismus—in agreement with other authors, defined by Garone et al. as lasting less than 30 days—is the most common ocular motility disorder causing an emergency visit during childhood, and brain malignancies are rare but an important differential diagnosis [3,4]. Vomiting, reduced consciousness, pupillary defects, gait abnormalities, and ptosis are red flags in a physical examination for this disorder.

AACE is defined as sudden-onset convergent strabismus with diplopia [5]. It shows an equal angle of deviation and has to be distinguished from infantile or accommodative esotropia [6]. Since the spontaneous recovery of AACE is the exception, therapeutic interventions using prism lenses or strabismus surgery are usually necessary [6,7,8]. The literature distinguishes as sub-types of this disorder AACE after monocular occlusion, possibly with pre-existing esophoria, an idiopathic form with low-degree hyperopia, and a typically progressive form with moderated myopia and esotropia greater at a distance than at a close range [6] Especially this latter so-called Bielschowsky type might be linked to an excess convergence tone associated with near work and, consequently, increased during the COVID-19 pandemic [6,8,9]. To be distinguished from these types is a recently repeatedly described subgroup of children with AACE who have an intracranial pathology (Table 1).

At present, no clear clinical guidelines or evidence-based decision making for acute-onset childhood strabismus including AACE exist, with the effect that there is controversy about the best evaluation approach [6,10]. Some authors emphasize the possibility of intracranial pathology as the cause of AACE and suggest considering MRI as a diagnostic screening tool [11,12]. In clinical practice, at least in many institutions, this results in a very high utilization of MRI in children presenting with recently established AACE. For such high-quality MRI in young infants, anesthesia—which carries a risk burden for the patient—is still necessary.

## 2. Methods

Data from patients diagnosed with strabismus from the University hospital in Leipzig, Germany, were analyzed 20 years retrospectively (2002–2022). We used “Data Warehouse” (electronic hospital database SAP SE, Walldorf, Germany) to identify all inpatients treated at the Leipzig University Department of Pediatrics (LDP) with an ICD-10 admission diagnosis of concomitant strabismus (ICD-10 H50.0 and H50.3) and the procedure of cranial MRI. Data were extracted from the patients’ digital medical records to identify cases of acute strabismus. A total of 54 patient records were identified, and 21 were included in this case series based on the following criteria: patients with acute-onset concomitant esotropia as the main diagnosis leading to cranial imaging. Patients with cranial nerve palsy incorrectly coded as concomitant strabismus at admission were excluded (e.g., paralytic strabismus should be coded as H49 according to ICD-10). Patients in whom the strabismus turned out to be long-standing or in whom the MRI was carried out due to other symptoms, i.e., the strabismus was only an accompanying symptom, were also excluded.

The local diagnostic algorithm for AACE included an orthoptic examination with special attention to ocular motility, the angle of strabismus for near and far targets, and incomitance in the vertical and lateral gazes including A and V patterns. Among others, the ophthalmologic assessment evaluated visual acuity, the anterior section of the eye, pupillary reactivity, macula, and optic disc. Additionally, a neuropediatric examination was performed for all the patients. Typically, cranial MRI was carried out, with the exception of cases with long-standing AACE, for which an arbitrary time frame was determined to be greater than 6 months, with a non-benign cause considered very unlikely in these cases. This mostly applied to patients who had been referred from other institutions for a second opinion.

The therapeutic algorithm consisted of attempting a hyperopic correction where indicated, prism lenses, and, if these measures failed, strabismus surgery.

Further, we compared our case series with cases from the literature and analyzed these cases in as much detail as possible with regard to the reported neurological and ophthalmological signs and symptoms in order to develop diagnostic recommendations in this overall context.

## 3. Results

Out of the 54 patients (100%), 21 patients (38%) had AACE. The age range was from 2 to 12 years; the mean age at the time of diagnosis was 5.5 years. According to the ophthalmological diagnostic algorithm, all the patients received a cranial MRI with normal results, except for one patient showing a highly parietal gliotic scar as an insignificant finding which was not causative for strabismus and had no therapeutic consequences (Table 1).

The AACE group presented with isolated strabismus, had no further signs or symptoms besides double vision, and had a normal neurological examination. Ultimately, all the patients maintained normal binocular function.

Our review of case series retrieved 347 cases, combined with our cases for a total of 368. In six cases—corresponding to 1.6%—a relevant intracranial pathology was found, including one case of medulloblastoma (Table 2) [11]. In three of these six cases, pathological clinical findings were detected that would have individually justified a cranial MRI. This statement also applies to the overview of individual cases (Table 3). We found insufficient clinical details reported in the other three cases to decide whether clinical detection would have been possible.

According to the literature, we classified the three cases of Chiari 1 malformation as having an unsafe causal relation to the AACE [6].

## 4. Discussion

The aim of this study was to collect data to estimate the risk of relevant intracranial pathology and thereby better assess the need for cranial imaging in children with acute acquired concomitant esotropia. For all our patients, the MRI never gave an indication of the need for acute neuropediatric or neurosurgical intervention.

Consequently, we conclude that neuroimaging is not necessary for patients with isolated AACE as a diagnostic screening tool. This is in line with Dotan et al., who included 20 patients in a retrospective study. The inclusion criteria in their study were as follows: patients with AACE without further neuropathological findings or ophthalmological abnormalities and who received an MRI. In that study, in 19 out of the 20 cases, the MRI was found to be normal; one child did not undergo an MRI and the type of strabismus was diagnosed to be benign after a long-term follow-up. These authors suggested the restrained use of cranial imaging in isolated strabismus [15]. This fits earlier reports testifying that, even in acute esotropia occurring after 5 years of age, an intracranial pathology is unlikely without other clinical signs or symptoms [7].

We also agree with Chong et al., who argued that isolated strabismus is not an indication for an MRI [10]. They described 70 pediatric patients who received neuroimaging due to strabismus over the last 10 years. A total of 75.7% of these patients had “isolated strabismus”, whereby the authors also included cases of strabismus caused by apparently isolated abducens nerve palsy into this category. In the whole group, concomitant esotropia was present in only 35 patients, and no pathological imaging findings were found in any patient with acute concomitant strabismus, supporting our statement [10]. Gilbert at al. summarized the existing literature on AACE and claimed that MRIs should be performed if nystagmus, abduction limitation, or optic nerve swelling are present or if neurological symptoms like headache, vertigo, imbalance, weakness, or nausea occur [6]. Considering the cases of Chiari I malformations, the above researchers derive, from their extensive literature analysis, its uncertain contribution to strabismus [6]. This view is also shared by Côté et al., who classify a Chiari I malformation in their series as a finding with an uncertain contribution to esotropia [11]. Despite individual case reports suggesting the opposite [20], in the presence of AACE, this malformation has no agreed therapeutic consequence and certainly not for urgent intervention.

The V pattern has been suggested in the literature as an ophthalmological indication of intracranial pathology [23]. It is at least noteworthy that this V pattern is described in one of the few cases of brain tumor in the case series.

We conclude that these case series support our findings, despite the following individual cases [21,22]:

Simon et al. reported a 4.5-year-old girl presenting with AACE and a cerebellar astrocytoma presenting in computed tomography. Related to the accompanying signs and symptoms, there were recurring headaches in the patient’s history and “mild dysmetria of all four extremities”. Interestingly, AACE persisted after tumor surgery. In our opinion, this underlines the importance of carful history taking and detailed neurological examination instead of primary brain imaging with anesthesia [21].

Astle et al. reported on a 15-year-old boy who presented with AACE, diplopia, and headaches for months that did not respond reliably to non-pharmacological or medicinal measures. A cerebellar tumor was seen on a cerebral CT scan. After removal, all the symptoms declined but residual esotropia persisted [22].

Considering such single cases in the literature of patients presenting AACE with only minor accompanying complaints or symptoms and intracranial pathology, we propose to set the indication generously in patients who present with headache, even slight coordination deficits, or similar subtle neurological signs or symptoms as well as a V pattern in orthoptic examination but to reject MRI as a routine measure for all children with AACE (Table 2 and Table 3).

In our opinion, the detailed analysis of these and other case reports underlines that, in the overwhelming majority, a thorough ophthalmological and neurological examination provides sufficient evidence of an intracranial pathology causing AACE.

## 5. Conclusions

AACE is often examined using brain imaging. The hereby presented data strongly argue against brain imaging as a routine screening procedure, especially if dependent on anesthesia. In our opinion, cranial MRI must be performed in patients with additional neurological findings or ophthalmologic abnormalities such as paralytic strabismus. In all other instances, a close follow-up seems appropriate.

## Figures and Tables

**Table 1:** MRI findings in AACE.

Number of Patients	Findings in MRI	Therapeutic Consequence
	1	none
21	gliotic scar	

**Table 2:** Review of AACE case series.

Study	Children with AACE (n/All Patients)	Patients with Findings in Cerebral Imaging (n)	Neurologic/Ophthalmic Findings	Cerebral Imaging Findings	Note
Clark et al., 1989 [5]	6/6	0	Diplopia	0	
Legmann Simon et al., 1997 [7]	9/10	1	Not documented	Chiari I malformation	Nine children, brain imaging in all but one
Lyons et al., 1999 [13]	10/10	1	Diplopia, V pattern, “apparently normal cerebellar function”	One cerebellar astrocytoma	
Buch et al., 2015 [12]	48/48	2	(1) Abnormal head position, within 2 weeks of presentation nystagmus, nausea, and vomiting (2) AACE at age 4 responding to spectacles, recurrence of AACE 5 years later	(1) Pontine glioma (2) “ill-defined thalamic lesion with no indication for intervention”	(2) Incidental finding in cMRI
Schörkhuber et al., 2018 [14]	53/53	2	(1) Nystagmus, bilateral papilledema (2) Not documented	(1) Sinus vein thrombosis (2) Mega cisterna magna, hydrocephalus	(2) Known congenital cerebral malformations
Dotan et al., 2020 [15]	20/20	0	0	-	One patient without MRI did not show neurological deficits in follow-up examination over 2 years
Lekskul et al., 2021 [8]	15/30	1	Headache, diplopia, nystagmus	Chiari I malformation	A total of 15 children and 15 adults. Only 43.3% of the patients received an MRI if neurological or ophthalmological symptoms were present
Chong et al., 2021 [10]	35/70	0	0	0	No pathological imaging findings in the 35 patients with acute concomitant strabismus
Meng et al., 2022 [16]	8/51	3	Not documented	(1) Pituitary tumor (2) Brain stem tumor (3) Chiari I malformation	A total of 8 children and 42 adults. Neurological history and examination not described; not clear whether isolated
Montriwet, 2023 [17]	36/41	0	0	0	36 children; 5 adults.
Côté et al., 2024 [11]	107/107	6	0	Five uncertain findings, one medulloblastoma	In the case of the medulloblastoma, no neurological details were given

**Table 3:** Overview of AACE case reports.

	Neurological Findings	Ophthalmological Findings	MRI Findings	Note	Age
Armenti et al., 2021 [18]	Headache	Horizontal jerk nystagmus in lateral gaze, differing amplitude R vs. L	Demyelination, thalamic lesion	Diagnosed with multiple sclerosis	16 years old
Lee et al., 2009 [19]	-	Papillary edema	Cerebellar tumor and hydrocephalus		3 years old
Defoort-Dhellemmes et al., 2002 [20]	Headache, diplopia	-	Chiari I malformation		9 years old
Simon et al., 1996 [21]	Headache, dysmetria	Esotropia increasing during 3 weeks from 30 prism to 45 prism diopter	Cerebellar astrocytoma	Tumor surgery did not heal esotropia	4.5 years old
Astle et al., 1994 [22]	Worsening headache	Diplopia	Cerebellar tumor		15 years old

## Data Availability

The data presented in this study are available on request from the corresponding and the senior author. The data are not publicly available due to privacy reasons.
